# Mind the gap: underreporting of key compartments in endometriosis MRI with free-text and non-disease-specific templates

**DOI:** 10.1186/s13244-026-02210-x

**Published:** 2026-02-09

**Authors:** Christian Deniffel, Gustav Andreisek, Egon Burian, Eliane Pauli, Matthias Oelke, Khashayar Namdar, Christian Houbois, Amelie Lutz, Dominik Deniffel

**Affiliations:** 1 Department of Diagnostic Radiology, Cantonal Hospital Frauenfeld, Spital Thurgau AG, Frauenfeld, Switzerland; 2 Faculty of Medicine, University of Zurich, Zurich, Switzerland; 3 Institute of Diagnostic and Interventional Radiology, University Hospital Zurich, Zurich, Switzerland; 4 Department of Obstetrics and Gynaecology, Cantonal Hospital Frauenfeld, Spital Thurgau AG, Frauenfeld, Switzerland; 5 Hannover Medical School, Hannover, Germany; 6 Institute of Medical Science, University of Toronto, Toronto, ON Canada; 7 Department of Medical Imaging, University of Toronto, Toronto, ON Canada; 8 Department of Medical Imaging, Sunnybrook Health Sciences Centre, ON Toronto, Canada; 9 TUM School of Medicine and Health, Technical University of Munich, Munich, Germany

**Keywords:** Endometriosis, Magnetic resonance imaging, Pelvis, Structured reporting, #Enzian

## Abstract

**Objectives:**

To evaluate the impact of different reporting approaches on the completeness of endometriosis documentation in pelvic MRI reports.

**Materials and methods:**

Retrospective single-center analysis of 186 pelvic MRI reports categorized as free-text (*n* = 102), general template (*n* = 24), or endometriosis-specific template (*n* = 60). Completeness was assessed for ten anatomical compartments based on the #Enzian classification. Rates were compared with Kruskal–Wallis test; compartment-level documentation was modeled with Firth’s penalized logistic regression adjusted for reporting bias from pathological findings; temporal trends were analyzed with multinomial logistic regression.

**Results:**

Report completeness differed significantly between report types (median 80.0% [IQR 22.5] for endometriosis-specific templates; 60.0% [20.0] for general templates; and 50.0% [20.0] for free-text; *p* < 0.0001). Compartment-level documentation for free-text was low for ureter (25.5%), peritoneum (25.5%), uterosacral ligaments (25.5%), fallopian tubes (33.3%) and vagina/rectovaginal space (45.1%); corresponding rates were 70.8%, 33.3%, 16.7%, 37.5%, 33.3% for general templates and 71.7%, 50.0%, 71.7%, 65.0%, 81.7% for endometriosis-specific templates. Endometriosis-specific templates yielded higher adjusted odds ratios (aOR) of documenting critical compartments than free-text, including bladder (aOR 12.8 [95% CI: 5.7–34.3]), rectum (6.5 [3.1–15.4]), uterus (5.9 [2.6–13.5]), vagina/rectovaginal space (5.4 [2.4–14.1]), uterosacral ligaments (3.1 [1.5–6.9]), and fallopian tubes (2.5 [1.2–5.2]). General templates showed inconsistent benefits, with deficiencies for key compartments (uterosacral ligaments: 0.2 [0.03–0.6]; fallopian tubes: 1.0 [0.4–2.6]; vagina/rectovaginal space: 0.6 [0.1–1.7]). Free-text reporting predominated throughout the 37-month observation period (58.5% at study end).

**Conclusions:**

Endometriosis-specific structured templates markedly improve documentation completeness versus general templates and free-text, with key compartments underreported in unstructured and generic structured formats.

**Critical relevance statement:**

By quantifying documentation gains of disease-specific MRI templates over generic structured and narrative formats, this study provides actionable evidence to implement targeted structured reporting to improve surgical planning and multidisciplinary communication in endometriosis.

**Key Points:**

- Endometriosis-specific MRI templates achieve higher documentation completeness compared to non-disease-specific templates and free-text reports.
- Disease-specific templates achieved 80% completeness versus 60% for general templates and 50% for free-text.
- Free-text reports underreport critical anatomical compartments, such as uterosacral ligaments, fallopian tubes and vagina/rectovaginal space.
- Endometriosis-specific templates showed up to 13-fold higher odds of documenting critical compartments versus free-text.
- Template specificity, not mere structure, drives comprehensive endometriosis reporting.

**Graphical Abstract:**

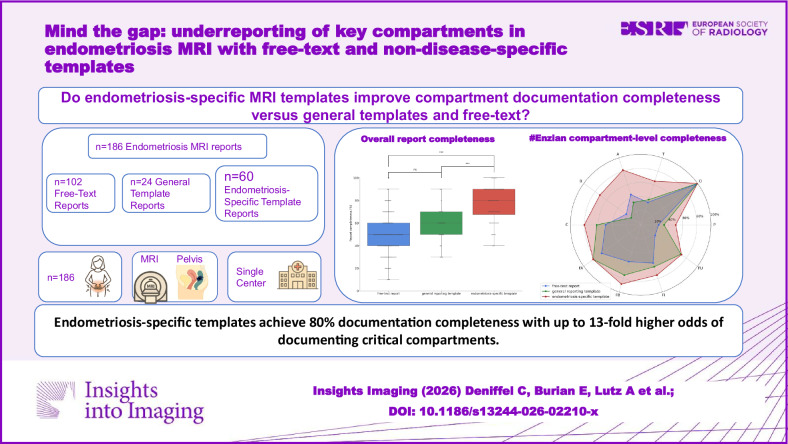

## Introduction

Endometriosis affects approximately 10% of women of reproductive age, leading to chronic pelvic pain, dysmenorrhea, and infertility, often requiring complex multidisciplinary surgery [1]. While laparoscopy has historically been considered the diagnostic gold standard, clinical practice has evolved toward non-invasive triage for deep endometriosis, with imaging now increasingly used for diagnosis and surgical planning [2].

A recent international intersociety consensus recommends transvaginal ultrasound as the first-line modality for suspected endometriosis, citing its good diagnostic accuracy, wide availability, and low cost. However, the consensus also acknowledges that ultrasound is less accurate, especially for deep infiltrating endometriosis involving the parametria and uterosacral ligaments [2]. The 2025 ESUR consensus recommends MRI as the preferred second-line modality for several clinical scenarios: when transvaginal ultrasound is inconclusive, when surgical treatment is planned, or if symptoms persist after surgery [3]. In these scenarios, MRI offers a comprehensive pelvic assessment and precise lesion mapping across anatomical compartments.

Recognizing the complexity of surgical management, multiple professional societies, including ESUR [3, 4], ESHRE [5], the Canadian Association of Radiologists (CAR) [6], and the French Health Agency [7], advocate for structured MRI reporting to optimize surgical planning and multidisciplinary communication.

Structured classification systems provide the framework for disease-specific structured reports. The #Enzian classification, an imaging adaptation of a surgical scoring system, divides endometriosis into standardized anatomical compartments: P (peritoneum), O (ovaries), T (tubo-ovarian), with deep infiltrating endometriosis compartments A (vagina/rectovaginal space), B (uterosacral ligaments/parametria), C (rectum), and extragenital sites FA (adenomyosis), FB (bladder), FI (intestine), FU (ureter), and Floc (other locations) [8]. The Deep Pelvic Endometriosis Index (dPEI) provides an alternative framework that organizes pelvic compartments with a focus on surgical complexity [9]. Both frameworks demonstrate good inter-reader agreement [10–12], though international consensus on a single scheme remains evolving. The 2024 International Consensus Statement, endorsed by multiple societies (including ISUOG, IDEA, ESGE, and ESHRE), recognizes #Enzian as the preferred system for anatomical mapping [2], supported by recent meta-analytic evidence demonstrating excellent diagnostic accuracy [13]. The 2025 ESUR consensus acknowledges both systems but identifies dPEI as the ‘most useful classification’ [3].

Despite strong professional endorsement, adoption of structured reporting in clinical practice remains inconsistent. A 2025 ESUR Delphi survey found that 85% of experts considered structured reports useful, yet only 55% used them regularly [3], reflecting broader implementation challenges across radiology [14].

Part of this gap may reflect the limited comparative empirical evidence underlying current expert consensus recommendations [3–7]. Jaramillo-Cardoso et al found that expert-structured reports increased sensitivity for deep endometriosis compared to narrative reports [15]. Similarly, Barbisan et al reported higher mention rates for critical compartments in structured versus free-text reports, though in a small, research-biased cohort [16]. Importantly, existing research in the field of structured reporting typically compares free-text with some sort of structured reporting, rather than evaluating different types of structured approaches [17].

Whether disease-specific templates outperform general pelvic templates, which provide organized layouts but lack compartment-specific content, remains unclear. Understanding how different levels of structure impact documentation completeness is therefore critical for evidence-based implementation strategies.

We hypothesized that endometriosis-specific MRI templates would yield higher compartment-level documentation completeness compared to both general pelvic templates and conventional free-text formats. Using the #Enzian compartment framework, we compared documentation rates across these three reporting approaches, adjusting for the presence of pathology, to provide quantitative guidance for template implementation initiatives.

## Materials and methods

### Study design and patient selection

This single-center, two-site retrospective study was approved by the local ethics board (Ethikkomission Ostschweiz, Project ID 2024-02242) as part of a larger project aimed at preparing datasets for artificial intelligence applications. Written informed consent was obtained from all participants. Cases were systematically identified through the Radiology Information System (RIS) by searching for MRI examinations performed using the specific institutional endometriosis protocols between January 2022 and February 2025. All cases identified through this search were included in the final analysis, resulting in 186 pelvic MRI reports. No additional exclusion criteria were applied.

### MRI protocol

All examinations were performed using standardized institutional MRI protocols for endometriosis, which included triplanar T2-weighted sequences of the small pelvis (4 mm slice thickness), T1-weighted Dixon sequences in axial and sagittal planes (4 mm slice thickness) acquired pre- and post-contrast, and large field-of-view coronal T2-weighted sequences including the kidneys. Examinations were conducted on either 1.5 T or 3 T MRI systems using phased array body coils.

### #Enzian classification system

The #Enzian classification system was used to categorize endometriosis findings across anatomical compartments. This system divides anatomical structures into several compartments [8]:

1. Main compartments: P (peritoneum), O (ovaries), T (tubo-ovarian condition).
2. Deep infiltrating endometriosis compartments: A (vagina/rectovaginal space), B (uterosacral ligaments/parametria), C (rectum).
3. Extragenital Endometriosis (F): FA (adenomyosis uteri), FB (bladder), FI (intestine - excluding rectum), FU (Ureter), Floc (other locations).

### Report review and evaluation

All eligible MRI reports were collected by a resident (C.D.) and manually reviewed by a subspecialty-trained urogenital radiologist (D.D.), assisted by a large language model (LLM, Claude Sonnet 3.5, Anthropic). Reports were evaluated for three aspects: completeness of documentation, presence of pathologic findings, and report type classification. The LLM was employed solely for auxiliary text parsing and initial extraction of explicit compartment documentation from report text. The LLM served as a time-efficient tool to screen the large volume of reports, highlighting compartment‑relevant phrases for human review and thereby complementing, rather than replacing, manual assessment. All final determinations of compartment documentation and presence or absence of pathology were made by human expert review. Completeness was assessed using a binary system (mentioned/not mentioned) for ten anatomical compartments corresponding to the #Enzian classification (P, O, T, A, B, C, FA, FB, FI, FU). Explicit mention of each compartment was required; general statements such as “no endometriosis in the pelvis” did not qualify as documentation of individual compartments. Both normal and pathological descriptions qualified as documentation (e.g., “normal ovaries” or “ovarian endometrioma” both indicated that compartment O was assessed). Each explicitly mentioned compartment contributed 10% to the overall completeness score, with a maximum score of 100%. Pathologic findings were classified according to #Enzian criteria. If a compartment was not specifically mentioned, it was classified as not pathologic.

Reports were categorized into three types based on structure:

1. **Free-text reports**: Unstructured narrative reports without predefined sections or standardized terminology for endometriosis findings.
2. **General structured templates:** Reports using institutional templates designed for general female pelvic MRI studies but lacking specific organizational structure or terminology for endometriosis assessment by anatomical compartment (Suppl. Table S1).
3. **Endometriosis-specific templates:** Reports incorporating standardized terminology and dedicated sections for each anatomical compartment relevant to endometriosis assessment (Suppl. Table S2).

### Statistical analysis

Statistical analyses were performed using R software (version 4.5.0; R Foundation for Statistical Computing, Vienna, Austria). Overall report completeness was compared between report types using the Kruskal–Wallis test, with post hoc pairwise comparisons performed using Dunn’s test and Holm–Bonferroni correction for multiple testing.

Individual anatomical compartment mentioning rates were analyzed using multivariable Firth’s penalized logistic regression [18], adjusting for the presence or absence of pathology to control for potential reporting bias. To account for clustering of compartments within patients, confidence intervals (CIs) were derived using stratified cluster bootstrap resampling (1000 iterations), where entire patient records were resampled within each report type stratum. Adjusted odds ratios (aORs) with 95% CIs are reported; an aOR greater than 1.0 indicates higher odds of mentioning a compartment relative to free-text reporting, independent of endometriosis presence. Firth’s penalized logistic regression was chosen to stabilize coefficient estimates in sparse data settings because some predictor–outcome combinations exhibited quasi-complete separation. In practical terms, this model estimates the independent effect of report format on compartment documentation by controlling for the inherent bias toward reporting pathological findings, while preventing model failure due to rare events.

Temporal trends in reporting practices were evaluated using multinomial logistic regression; 95% CIs were computed via bootstrapping (1000 iterations). The time variable (months since study start) was modeled with a range of functional forms, including linear, logarithmic, quadratic, exponential, cubic polynomial, and natural cubic splines (up to four knots at quantiles). Model selection was based on the Bayesian Information Criterion (BIC), which balances model fit with complexity by penalizing overly complex models. The model yielding the lowest BIC was retained for inference.

For analyses involving bootstrap CIs, statistical significance was inferred when the 95% CI excluded the null value (1.00 for odds ratios, 0 for regression coefficients); *p*-values are not separately reported in these cases. For other analyses, a *p*-value < 0.05 was considered statistically significant.

## Results

### Study population

A total of 186 pelvic MRI reports from patients with suspected or confirmed endometriosis were included. One hundred and two reports (54.8%) were free-text, 24 (12.9%) used a general pelvic MRI template, and 60 (32.3%) employed an endometriosis-specific template. Pathological findings were most common in the ovaries (O; 30.6%), followed by the uterus/adenomyosis (FA; 25.8%) and the vagina/rectovaginal space (A; 19.4%) (Table 1).

**Table 1:** Characteristics of patients by report type

Characteristics	Report type		
	Free-text report	General reporting template	Endometriosis-specific template
Patients (*N*)	102	24	60
Time period of the MRI exam	02/2022–02/2025	05/2022–02/2025	01/2022–01/2025
Distinct reporters (*N*)	36	15	21
Patient’s age (years)	36.5 (8.1)	36.2 (10.3)	36.5 (9.2)
Pathologic findings (per #Enzian compartment)			
P	9 (8.8%)	0 (0.0%)	9 (15.0%)
O	29 (28.4%)	6 (25.0%)	22 (36.7%)
T	18 (17.6%)	1 (4.2%)	11 (18.3%)
A	16 (15.7%)	3 (12.5%)	17 (28.3%)
B	14 (13.7%)	2 (8.3%)	15 (25.0%)
C	7 (6.9%)	2 (8.3%)	6 (10.0%)
FA	25 (24.5%)	5 (20.8%)	18 (30.0%)
FB	1 (1.0%)	0 (0.0%)	4 (6.7%)
FI	9 (8.8%)	0 (0.0%)	4 (6.7%)
FU	1 (1.0%)	0 (0.0%)	1 (1.7%)
Floc	2 (2.0%)	0 (0.0%)	1 (1.7%)

Statistics presented: *N* (%); mean (SD)

#Enzian compartment dictionary: P: Peritoneum; O: Ovaries; T: Tubo-ovarian; A: Vagina/Rectovaginal space; B: Uterosacral ligaments/Parametria; C: Rectum; FA: Adenomyosis uteri; FB: Bladder; FI: Intestine; FU: Ureter; Floc: Other extragenital locations

### Overall report completeness

**Fig. 1:**
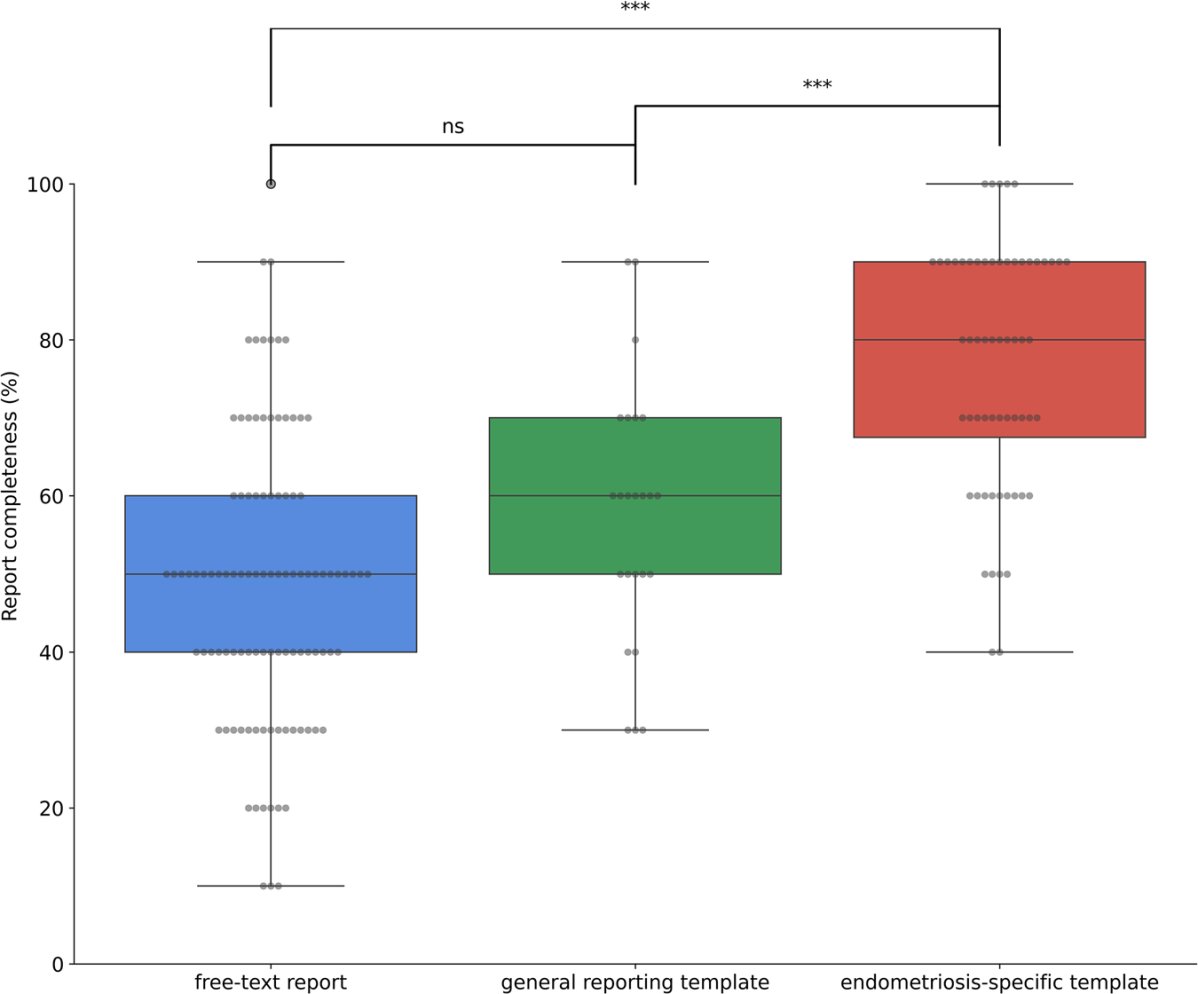
Distribution of report completeness by report type. Box plots showing the percentage of documented #Enzian compartments for free-text (*n* = 102), general template (*n* = 24), and endometriosis-specific template (*n* = 60) reports. Statistical significance is indicated by asterisks: ns not significant, *** *p* < 0.001 (Kruskal–Wallis test with post hoc Dunn’s test and Holm–Bonferroni correction)

Report completeness differed significantly between the three approaches (Kruskal–Wallis H = 66.13, *p* < 0.0001; Fig. 1). Median completeness was 80.0% (IQR 22.5%) for endometriosis-specific templates, 60.0% (IQR 20.0%) for general templates, and 50.0% (IQR 20.0%) for free-text reports. Dunn–Holm post hoc testing showed no significant difference between free-text and general templates (*p* = 0.051), but higher completeness for endometriosis-specific templates vs free-text (*p* < 0.0001) and vs general templates (*p* < 0.001).

### Mentioning rates by #Enzian compartment

**Fig. 2:**
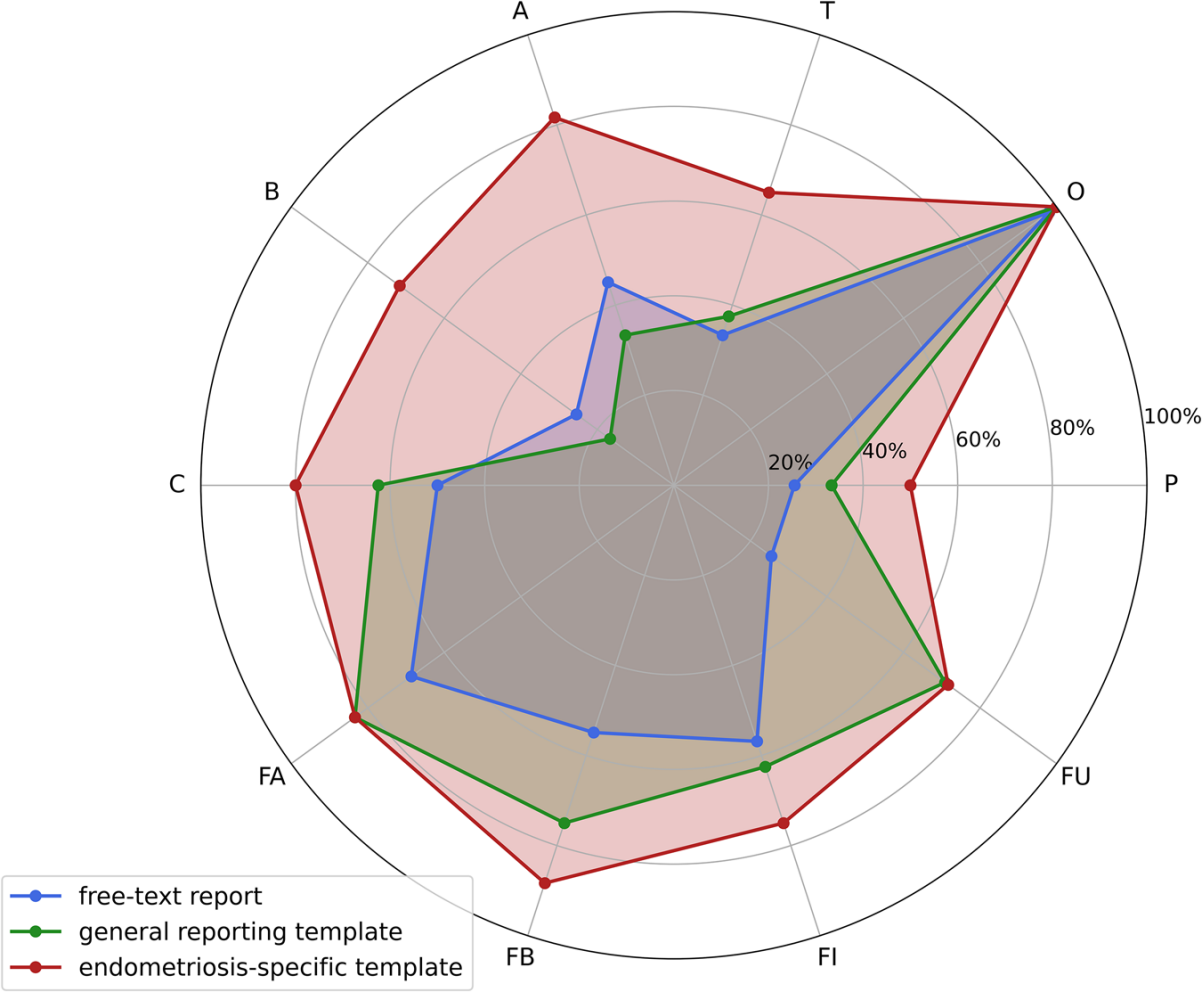
Radar plot comparing completeness rates across #Enzian compartments across report types. Documentation completeness for ten anatomical compartments is shown for free-text reports (blue), general structured templates (green), and endometriosis-specific templates (red). The radar plot demonstrates consistently higher mentioning rates in endometriosis-specific templates across most compartments, with particularly pronounced differences for compartments B and A (deep infiltrating endometriosis affecting uterosacral ligaments/parametria and rectovaginal space) and compartment T (tubo-ovarian adhesions), all critical for determining surgical complexity. #Enzian compartment dictionary: P: Peritoneum; O: Ovaries; T: Tubo-ovarian; A: Vagina/Rectovaginal space; B: Uterosacral ligaments/Parametria; C: Rectum; FA: Adenomyosis uteri; FB: Bladder; FI: Intestine; FU: Ureter; Floc: Other extragenital locations

Compartment-level completeness varied markedly between report types (Fig. 2, Suppl. Fig. S1).

For the ureter (FU) documentation rates were 25.5% (95% CI 17.6–34.3%) in free-text reports, 70.8% (50.0–87.5%) in general templates, and 71.7% (60.0–81.7%) in endometriosis-specific templates.

Peritoneum (P) was mentioned in 25.5% (17.6–36.3%) of free-text reports, 33.3% (16.7–54.2%) of general templates, and 50.0% (38.3–63.3%) of endometriosis-specific templates.

Uterosacral ligaments/parametria (B) were documented in 25.5% (17.6–34.3%) of free-text reports, 16.7% (4.2–36.7%) of general templates, and 71.7% (60.0–81.7%) of endometriosis-specific templates.

For the fallopian tubes (T), the corresponding rates were 33.3% (24.5–43.1%), 37.5% (20.8–58.3%) and 65.0% (51.7–75.0%).

Vagina/rectovaginal space (A) was documented in 45.1% (35.3–54.2%) of free-text reports, 33.3% (16.7–54.2%) of general templates, and 81.7% (71.7–90.0%) of endometriosis-specific templates.

Ovarian documentation (O) remained consistently high across all report types: 98.0% [93.1–100.0%] for free-text reports, 100.0% for general templates, and 100.0% for endometriosis-specific templates.

### Pathology-adjusted analysis

**Fig. 3:**
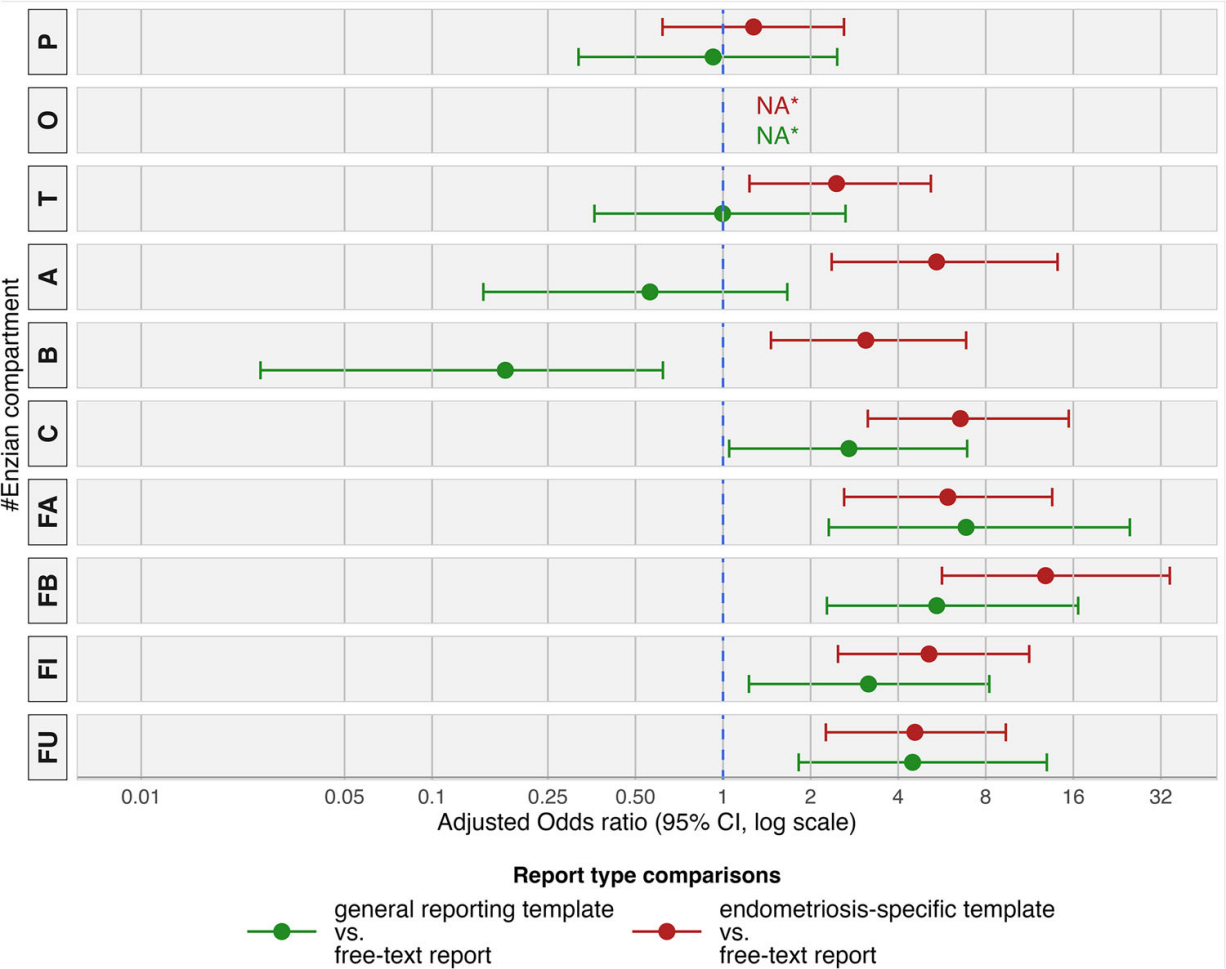
Forest plot showing adjusted odds ratios (aORs) for documentation completeness by anatomical #Enzian compartment and reporting approach. The plot displays the odds of explicit documentation for each compartment with 95% confidence intervals, comparing endometriosis-specific templates vs free-text reports (red) and general templates vs free-text reports (green). An aOR greater than 1.0 indicates higher odds of compartment documentation relative to free-text reporting. ORs were adjusted for pathological status (accounting for the inherent bias that pathological compartments are more likely to be documented) and patient clustering using Firth’s penalized logistic regression with stratified cluster bootstrap resampling. * Missing values (NA) for compartment O reflect model instability owing to near-complete documentation rates across all reporting approaches. #Enzian compartment dictionary: P: Peritoneum; O: Ovaries; T: Tubo-ovarian; A: Vagina/Rectovaginal space; B: Uterosacral ligaments/Parametria; C: Rectum; FA: Adenomyosis uteri; FB: Bladder; FI: Intestine; FU: Ureter; Floc: Other extragenital locations

Multivariable logistic regression analysis was employed to adjust for non-pathological findings, which were less likely to be documented regardless of report type (Suppl. Fig. S2). The odds of complete documentation were substantially higher with endometriosis-specific templates vs free-text reports across most anatomical compartments, except for compartment P (peritoneum: aOR 1.3, 95% CI: 0.6–2.6) and compartment O, where statistical analysis was limited due to perfect or near-perfect documentation across all report types (Fig. 3).

Significantly increased aORs for endometriosis-specific templates compared to free-text reports ranged from 2.5 (1.2–5.2) for compartment T (fallopian tubes) to 12.8 (5.7–34.3) for compartment FB (bladder). Notably high aORs were also observed for compartments C (rectum: 6.5 [3.1–15.4]), FA (uterus/adenomyosis: 5.9 [2.6–13.5]), A (vagina/rectovaginal space: 5.4 [2.4–14.1]), FI (intestine: 5.1 [2.5–11.3]), FU (ureter: 4.6 [2.3–9.4]), and B (uterosacral ligaments/parametria: 3.1 [1.5–6.9]).

General templates showed inconsistent associations with documentation completeness compared to free-text reports, with non-significant associations for compartments P (peritoneum: 0.9 [0.3–2.5]), T (fallopian tubes: 1.0 [0.4–2.6]), and A (vagina/rectovaginal space: 0.6 [0.1–1.7]), and significantly reduced odds for compartment B (uterosacral ligaments/parametria: 0.2 [0.0–0.6]).

### Time trends in reporting practices

Analysis of reporting patterns over the study period from January 2022 to February 2025 was conducted using multinomial logistic regression; a logarithmic transformation of the time variable yielded the best model fit (Suppl. Table S1).

**Fig. 4:**
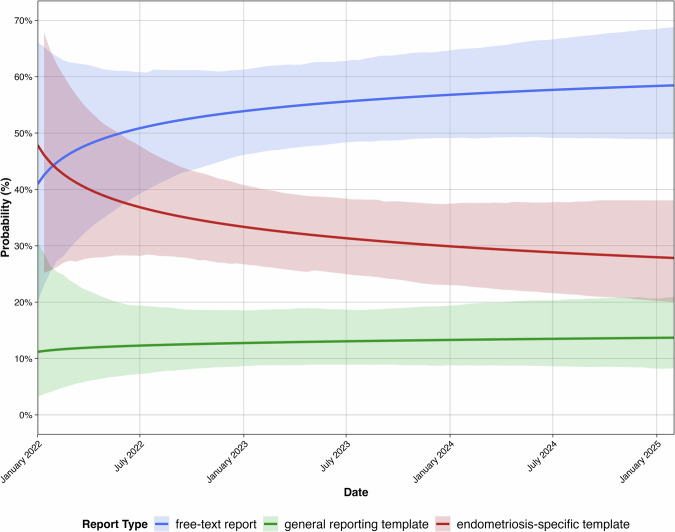
Temporal trends in reporting approach utilization from January 2022 to February 2025. The graph displays the predicted probability (*y*-axis) of each report type being utilized over time (*x*-axis, months since study start). Predicted probabilities were derived from multinomial logistic regression with logarithmic time transformation and bootstrapped 95% confidence intervals (shaded areas). Free-text reporting remained the predominant reporting format throughout the observation period (58.5% at study end)

No significant temporal trend in reporting practices was detected (general templates β = −0.03, 95% CI −0.91 to 1.42; endometriosis-specific templates β = −0.26, [−0.67 to 0.13] vs free-text). Predicted probabilities for study end were 58.5% (49.0–68.8%) for free-text, 13.7% (8.3–20.9%) for general templates and 27.8% (20.0–38.1%) for endometriosis-specific templates, corresponding to odds ratios of 9.14 (4.01–20.73) vs general templates and 3.69 (1.66–8.75) vs endometriosis-specific templates for issuing a free-text report (Fig. 4).

## Discussion

Endometriosis-specific structured templates achieved 80% documentation completeness across #Enzian compartments, clearly outperforming both general templates (60%) and free-text reports (50%). After adjusting for pathological findings, disease-specific templates raised the odds of documenting critical compartments by up to 13-fold compared with free-text, while general templates showed inconsistent or negative benefits for critical structures like uterosacral ligaments, fallopian tubes and the rectovaginal space.

This study provides the first quantitative proof that template specificity, not structure alone, determines documentation quality in endometriosis MRI. General templates left persistent documentation gaps for key compartments, challenging assumptions that any structured approach suffices and validating the need for targeted disease-specific frameworks as advocated by recent consensus statements [2, 3]. By quantifying effect sizes across three reporting approaches in a real-world clinical cohort, our work extends prior expert consensus [2, 3] and smaller pilot studies [15, 16] and offers actionable evidence for departments deciding whether to adapt existing templates or implement disease-specific solutions.

Only one prior study assessed compartment-level completeness in endometriosis MRI. Barbisan et al had two radiologists re-dictate 28 MRI cases in both structured and unstructured formats, reporting rectum in 61%/96%, uterosacral ligaments in 71%/96%, vagina in 14%/39%, ureter in 4%/75%, ovaries in 93%/100%, and bladder in 54%/100% of narrative reports [16]. In our 186-patient cohort, free-text reports from 36 radiologists showed similarly high ovarian coverage and comparable rates for vagina/rectovaginal septum (45%), ureter (26%), and bladder (55%), but lower mention of uterosacral ligaments (26%) and rectum (50%). Barbisan et al’s study design likely introduced systematic completion bias via recall and Hawthorne effects. By analyzing original clinical reports from a large, diverse group of radiologists, our study provides pragmatic estimates less susceptible to research-induced bias. Since compartments containing visible endometriosis are inherently more likely to be documented than those appearing normal, regardless of reporting format, we adjusted for pathological status to avoid confounding by disease burden. This adjustment isolates the independent effect of report format on documentation completeness.

It is important to distinguish between documentation completeness, the systematic reporting of anatomical compartments, and diagnostic accuracy, the correct identification of pathological findings. Our endpoint captured whether compartments were explicitly mentioned in the report text, not whether all pathological findings were correctly identified. Consequently, an unmentioned compartment could reflect either an unassessed or overlooked site, or a compartment that was assessed and considered normal but not explicitly documented. Clinically, this ambiguity matters: without explicit documentation, treating gynecologists and surgeons cannot distinguish between ‘assessed and normal’ and ‘not assessed,’ which may affect surgical planning. While we can only speculate on the underlying reasons, it is striking that some of the least-documented sites in our series, parametria, uterosacral ligaments and fallopian tubes, are precisely those recognized as technically challenging for MRI assessment, with lower imaging sensitivity and evolving anatomical terminology [2, 3, 13]. By contrast, anatomically familiar structures like the ovaries showed near-universal documentation (98–100%) across all report types. Disease-specific templates may improve diagnostic pathways by prompting systematic compartment-by-compartment assessment and explicit documentation. Prior work suggests that expert-structured reporting may improve sensitivity for deep endometriosis [15], but structural improvements in reporting alone may not necessarily alter clinical management [19]; prospective studies directly comparing diagnostic accuracy and downstream clinical outcomes across reporting formats therefore remain needed. Accordingly, our findings primarily inform reporting quality and communication, rather than diagnostic performance.

Despite guideline endorsement [3–6] and template availability, free-text predominated throughout our 37-month study (58.5% at study end), mirroring international adoption rates below 60% [3, 20]. The ESR’s 2023 position paper identifies multiple barriers to structured reporting adoption: radiologists perceive templates as time-consuming, commonly used graphical check-box interfaces divert attention from images, and free-text offers narrative flexibility that many prefer for complex cases [14, 21]. Our experience suggests additional hurdles: the availability of multiple template options (general vs disease-specific) may paradoxically reduce adoption if radiologists are uncertain which template to use for a given indication. Furthermore, inadequate training or a lack of awareness about the clinical value of structured reporting may limit motivation for change. In the absence of institutional mandates, radiologists may therefore continue familiar free-text practices.

Overcoming these barriers requires institutional commitment and a clear focus on the practical benefits of disease‑specific templates. They should supplement rather than disrupt workflow, with mandatory training on the underlying anatomical framework and audit feedback on documentation gaps, as implemented in our study, to support sustained adoption. In this context, endometriosis‑specific templates act not only as a structured format but also as a cognitive “mind map” that guides radiologists through all relevant #Enzian compartments, including those our data identified as most frequently underreported in free‑text and general templates. At the same time, disease‑specific templates generate machine‑readable data for research and automated audits, mitigating the risk that future automated tools will perpetuate existing documentation gaps. Looking forward, large language models (LLMs) may offer a paradigm shift by extracting structured compartment data from free-text dictation, reconciling structured data capture with radiologists’ preference for narrative reporting [22].

Several limitations merit consideration. Our endpoint was explicit compartment documentation rather than diagnostic accuracy; therefore, this study cannot determine whether improved completeness translates into improved diagnostic accuracy. Given the inherent structural differences between report types, the reviewing radiologist was not blinded to the report type. However, potential observer bias was mitigated by the use of an LLM for initial text screening, which provided a standardized, report‑agnostic review layer. Our use of #Enzian as an audit framework does not address ongoing debates between classification systems, which were outside our study scope. At our institution, #Enzian-based anatomical mapping is mandatory for all endometriosis MRI reports for surgical planning. This institutional requirement, combined with the 2024 International Consensus endorsement [2] and recent meta-analytic validation [13], informed our decision to use #Enzian as the reference standard for assessing report completeness.

Endometriosis-specific templates raised documentation completeness from 50% to 80% with up to 13-fold higher odds of mentioning critical compartments, decisively outperforming both general templates and narrative reports. These findings provide evidence-based justification for institutional adoption of disease-specific structured reporting to close documentation gaps and align radiological practice with multidisciplinary surgical planning requirements.

## Supplementary information

Supplementary Material

## Data Availability

The datasets generated and/or analyzed during the current study are available from the corresponding author on reasonable request, subject to ethical and privacy regulations.

## Abbreviations

#Enzian: Imaging-adapted surgical endometriosis classification system
aOR: Adjusted odds ratio
BIC: Bayesian information criterion
CI: Confidence interval
ESHRE: European Society of Human Reproduction and Embryology
ESUR: European Society of Urogenital Radiology
IQR: Interquartile range
LLM: Large language model
