# Supplementary material for: Mind the gap: underreporting of key compartments in endometriosis MRI with free-text and non-disease-specific templates
Source: Insights Imaging. 2026 Feb 9;17:34. doi: 10.1186/s13244-026-02210-x (PMC12886655; doi:10.1186/s13244-026-02210-x)
Supplement: Supplementary file 1 — Supplementary Material [file 13244_2026_2210_MOESM1_ESM.pdf]

Mind the Gap: Underreporting of Key Compartments in Endometriosis MRI with Free-Text and Non-Disease-Specific Templates

ELECTRONIC SUPPLEMENTARY MATERIAL

Supplemental Table S1. Institutional general/non-disease specific female pelvis MRI reporting template

Uterus and adnexa:	
Bladder:	
Gastrointestinal tract (visualized segments):	
Peritoneum:	
Lymph nodes:	
Vessels:	
Skeleton / Soft tissues:	

Supplemental Table S2. Institutional endometriosis-specific female pelvis MRI reporting template

**Anterior Compartment:**	
Bladder:	
Ureters:	
Vesicouterine pouch:	
Vesicovaginal space:	
Prevesical space:	
**Middle Compartment:**	
Ovaries:	
Fallopian tubes:	
Uterus:	
Uterine ligaments:	
Vagina:	
**Posterior Compartment:**	
Rectum:	
Retrocervical space:	
Rectovaginal space:	
Uterosacral ligaments:	
**Additional locations:**	
Colon / Appendix / Small bowel:	
Neural structures (particularly the lumbosacral plexus):	
**Miscellaneous:**	
Lymph nodes:	
Vessels:	
Skeleton / Soft tissues:	

Supplemental Table S3. Comparison of Multinomial Regression Models

Model	AIC	BIC
Logarithmic	362.4856	375.3886
Exponential	364.1016	377.0046
Linear	364.1632	377.0662
Quadratic	366.4283	385.7828
Cubic	360.1493	385.9552
Spline_Q50	367.0030	386.3575
Spline_Q33,67	361.7517	387.5577
Spline_Q10,50,90	362.1245	394.3820
Spline_Q5,35,65,95	365.8640	404.5730

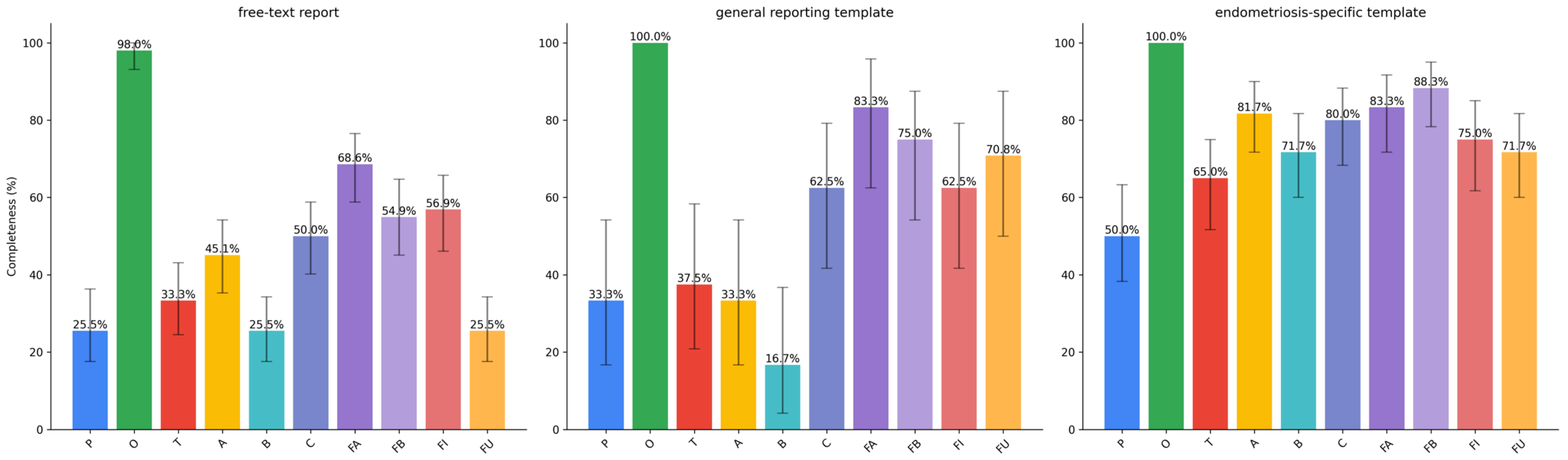

Supplemental Figure S1. Documentation completeness by #Enzian compartment and report type. Bars depict the percentage of reports mentioning each compartment in free-text reports (blue), general reporting templates (green) and endometriosis-specific templates (red); whiskers indicate bootstrapped 95 % confidence intervals. #ENZIAN compartment dictionary: P: Peritoneum; O: Ovaries; T: Tubo-ovarian; A: Vagina/Rectovaginal space; B: Uterosacral ligaments/Parametria; C: Rectum; FA: Adenomyosis uteri; FB: Bladder; FI: Intestine; FU: Ureter; Floc: Other extragenital locations.

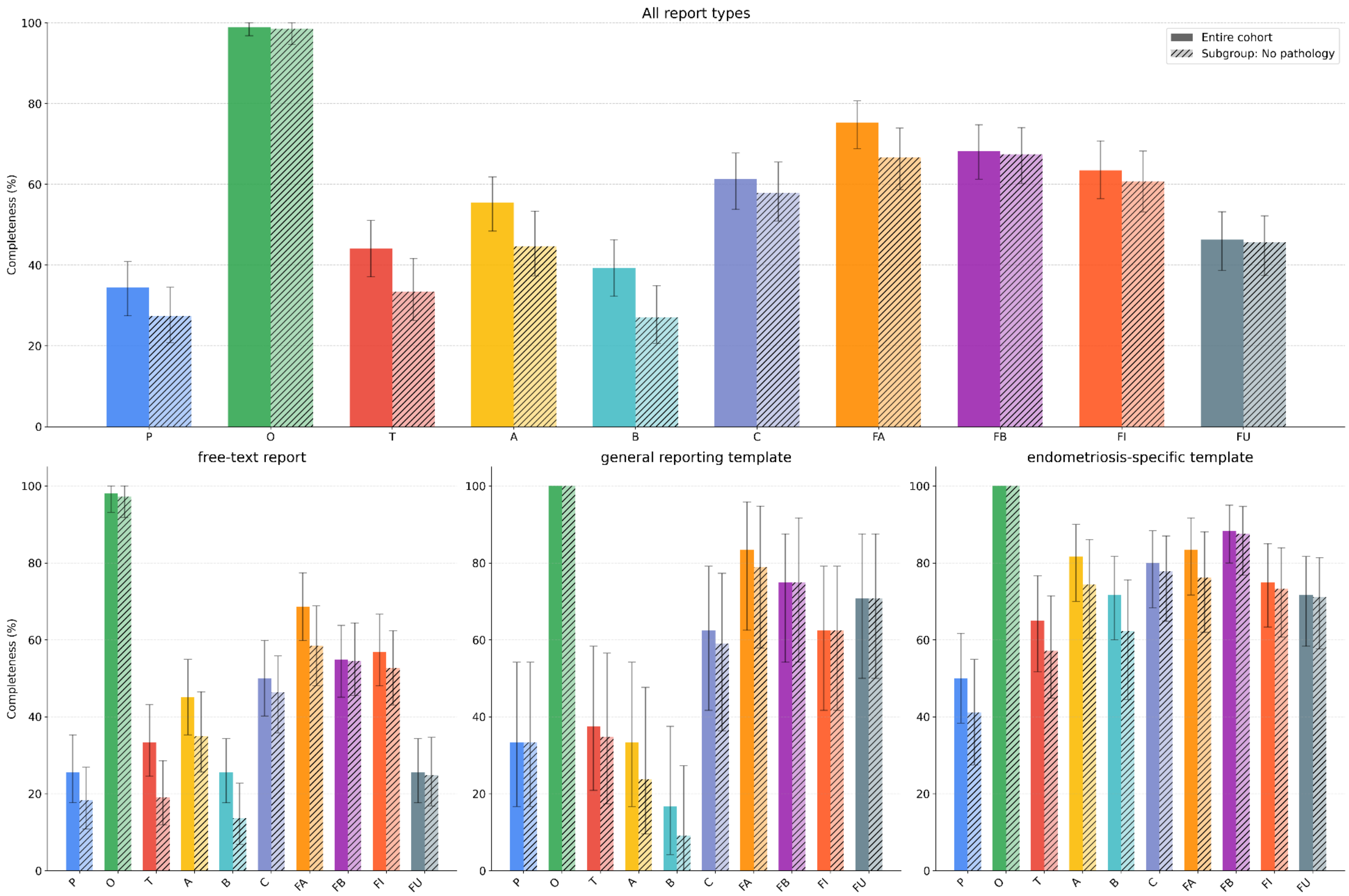

Supplemental Figure S2. Documentation completeness by #ENZIAN compartment in the entire cohort (solid bars) and in the subgroup without pathological findings in the respective compartment (hatched bars), displayed overall and separately for free-text reports, general templates, and endometriosis-specific templates. Whiskers represent bootstrapped 95 % confidence intervals. #ENZIAN compartment dictionary: P: Peritoneum; O: Ovaries; T: Tubo-ovarian; A: Vagina/Rectovaginal space; B: Uterosacral ligaments/Parametria; C: Rectum; FA: Adenomyosis uteri; FB: Bladder; FI: Intestine; FU: Ureter; Floc: Other extragenital locations.
